# Biochemical studies on sphingolipids of *Artemia franciscana*: complex neutral glycosphingolipids

**DOI:** 10.1007/s10719-012-9436-8

**Published:** 2012-08-14

**Authors:** Hisao Kojima, Yukako Tohsato, Kazuya Kabayama, Saki Itonori, Masahiro Ito

**Affiliations:** 1 Institute of Glycoscience, Tokai University, 4-1-1 Kitakaname, Hiratsuka, Kanagawa 259-1292 Japan; 2 Department of Chemistry, Faculty of Liberal Arts and Education, Shiga University, 2-5-1 Hiratsu, Otsu, Shiga 520-0862 Japan; 3 Department of Bioinformatics, College of Life Sciences, Ritsumeikan University, 1-1-1 Nojihigashi, Kusatsu, Shiga 525-8577 Japan

**Keywords:** Sphingosine, Fucomannolipid, Terminal α-*N*-acetylglucosamine residue, Structure characterization, Branchiopoda, Dormant cyst

## Abstract

Brine shrimp are primitive crustacean arthropodal model organisms, second to daphnia, which can survive in high-salinity environments. Their oviposited cysts, cuticle-covered diapausing eggs, are highly resistant to dryness. To elucidate specialties of brine shrimp, this study characterized glycosphingolipids, which are signal transduction-associated material. A group of novel and complex fucosyl glycosphingolipids were separated and identified from cysts of the brine shrimp *Artemia franciscana* by repeated lipid extraction, alkaline methanolysis, acid treatment, successive column chromatography, and post-source decay measurements by matrix-assisted laser desorption/ionization time-of-flight mass spectrometry. Structures of the glycosphingolipids were elucidated by conventional structural characterization and mass spectrometry, and the compounds were identified as GlcNAcβ1-3GalNAcβ1-4(GlcNAcα1-2Fucα1-3)GlcNAcβ1-3Manβ1-4Glcβ1-Cer, GalNAcβ1-4(Fucα1-3)GlcNAcβ1-3GalNAcβ1-4(GlcNAcα1-2Fucα1-3)GlcNAcβ1-3Manβ1-4Glcβ1-Cer, and GalNAcβ1-4(GlcNAcα1-2Fucα1-3)GlcNAcβ1-3GalNAcβ1-4(GlcNAcα1-2Fucα1-3)GlcNAcβ1-3Manβ1-4Glcβ1-Cer. These compounds also contained a branching, non-arthro-series disaccharide with an α-GlcNAc terminus, similar to that found in a previously reported ceramide hexasaccharide (III^3^(GlcNAcα2Fucα)-At_4_Cer). The glycans within these complex GSLs are longer than reported glycans of the animal kingdom containing α-GlcNAc terminus. These complex GSLs as well as the longest GSL with ten sugar residues, ceramide decasaccharide (CDeS), contain the fucosylated LacdiNAc sequence reported to associate with parasitism/immunosuppression and the α-GlcNAc terminus reported to show a certain antibacterial effect in other reports. CDeS, the longest GSL of this species, was found in the highest amount, which indicates that CDeS may be functionally important.

**Electronic supplementary material:**

The online version of this article (doi:10.1007/s10719-012-9436-8) contains supplementary material, which is available to authorized users.

## Introduction

Glycosphingolipid (GSL) is an amphipathic compound consisting of a sugar chain and a ceramide composed of a fatty acid and sphingoid base. GSLs are ubiquitous on the outer surface of the plasma membrane in animal cells, aggregated into patches called microdomains, and play an essential role in intercellular interaction and recognition [1–3]. However, a comprehensive understanding of GSL function has not yet been attained because of the structural complexity of the sugar chain and ceramide moiety [4, 5].

The study of invertebrate GSLs increases our understanding of human GSLs, helps determine which types of sphingolipids are essential for living animals, and clarifies evolutionary relationships between phyla in the animal kingdom. In the phylum Arthropoda, structural analyses of GSLs have been performed with flies [6, 7], and a characteristic arthro-series sugar chain (GlcNAcβ3Manβ4GlcCer; At_3_Cer) has been characterized. Functional analyses of GSLs by knockout experiments of *egghead* and *brainiac* have shown that At_3_Cer is essential for insect development [8]. Furthermore, At_3_Cer was detected in flies, including *Lucilia caesar* and *Calliphora vicina*, as well as in arthropods (the millipede *Parafontaria laminata armigera*) and crustaceans (*Euphausia superba*, *Macrobrachium nipponense*) [5]. We have further demonstrated the existence of At_3_Cer and some shorter GSLs, including GlcCer (CMS), mactosylceramide (MacCer, CDS), II^3^Fucα-MacCer (nonarthro-CTS), At_4_Cer (CTeS), II^3^(GlcNAcα2Fucα)-MacCer (nonarthro-CTeS), III^3^Fucα-At_4_Cer (CPS), and III^3^(GlcNAcα2Fucα)-At_4_Cer (CHS), in cysts of the brine shrimp *Artemia franciscana* [9]. However, it is more difficult to identify structural aspects of the sugar chain in complex GSLs than it is in shorter GSLs. Identifying the sugar chain in certain complex GSLs, which may be mixed with GSLs bound to similar oligosaccharides, depends on faint differences in polarity to determine long sugar sequences and the positions of substituted sugar residues at branching points. Several relatively complicated strategies have been employed to identify the structures of complex neutral GSLs, such as successive enzymatic degradation with exoglycosidase, chemical degradation to liberate partial oligosaccharides followed by Gas-liquid chromatography (GC) analysis, and fast atom bombardment (FAB) mass analysis of permethylated GSL to elucidate sugar sequences. In fact, there are relatively few reports that detail the structural analyses of complex GSLs in invertebrates. Complex GSLs have been identified in the green-bottle fly *L. caesar* and the blowfly *C. vicina* [10, 11], the human blood fluke *Schistosoma mansoni* [12–14], the bivalves *Corbicula sandai* [15] and *Hyriopsis schlegeli* [16], the seawater bivalve *Meretrix lusoria* [17], and the oyster *Ostrea gigas* [5].

The current study uses post-source decay (PSD) measurements coupled with matrix-assisted laser desorption/ionization time-of-flight mass spectrometry (MALDI-TOF MS) to analyze the structures of complex GSLs in diapausing eggs (cysts) of the brine shrimp *A. franciscana*, a crustacean arthropod harvested from the Great Salt Lake. These complex GSLs contain seven to ten sugar chains and exhibit a hybrid structure of core arthro-series sugar chains with a branching non-arthro-series disaccharide (GlcNAcα2Fucα).

## Materials and methods

### Separation of ceramide heptasaccharide (CHpS_1_, CHpS_2_), ceramide octasaccharide (COS_1_), and ceramide decasaccharide (CDeS)

Neutral GSL fractions were prepared as previously reported [9] and applied to a column of Iatrobeads (6RS-8060, Mitsubishi Kagaku Iatron Inc. Tokyo). The neutral GSLs were eluted with two linear gradient elution systems of C/M/W with compositions from 80:20:1 (v/v/v, 255 mL) to 50:50:5 (v/v/v, 325 mL) and from 50:50:5 (v/v/v, 252 mL) to 20:80:10 (v/v/v, 330 mL). Three-milliliter fractions were collected in each tube, and aliquots from every third tube were analyzed by high-performance thin-layer chromatography (HPTLC). Fractionated GSLs were analyzed by MALDI-TOF MS. CHpSes are denoted as CHpS_1_ and CHpS_2_ according to their order of elution, respectively. Similarly, COSes are denoted as COS_1_ and COS_2_.

### Separation of COS_2_ and ceramide nonasaccharide (CNS)

For isolation of COS_2_ and CNS, an independently prepared neutral GSL fraction (88 mg) was applied to an Iatrobeads column (ϕ1.0 × 56 cm). The neutral GSLs were eluted by a stepwise gradient elution system with one column volume each (44 mL) of pure chloroform, C/M 9:1 (v/v), C/M/W 80:20:1 (v/v/v), 70:30:3, 60:40:4, 50:50:5, 40:60:6, 30:70:7, 20:80:8, 10:90:9, and methanol/water 1:1 (v/v). The C/M/W 20:80:8 fraction including COS_2_ and CNS was further separated on a HPLC system (Shimadzu LC-10A) with an Iatrobeads column (ϕ1.0 × 55 cm) and eluted with a 75:30:5 (v/v/v) mixture of 1-propanol:water:28 % ammonium hydroxide at a flow rate of 1 mL/min. Three-milliliter fractions were collected in each tube, and aliquots from every third tube were tested by HPTLC. To remove silicic acid from the ammonium column, a brief purification with a Sep-pak C18 cartridge was done as follows: dried crude COS_2_ and CNS were dissolved in 2 mL of methanol, added to 6 mL of 50 mM NaCl, and applied to a Sep-pak C18 cartridge equilibrated with 25 mL each of water, methanol, and 50 mM NaCl, respectively. The cartridges were desalted with 30 mL of water, and the purified COS_2_ and CNS were eluted by 10 mL of methanol and dried under a nitrogen stream.

### Solvent system for thin-layer chromatography (TLC)

The following solvent systems were used for TLC: C/M/W (60:40:10, v/v/v and 55:45:10, v/v/v) and 1-propanol:water:28 % ammonium hydroxide (1-PrOH/W/NH_4_OH) (70:30:5, v/v/v). After QAE-Sephadex and Florisil column chromatography, the eluates were visualized on silica gel 60 TLC plates (Merck KGaA, Germany) by spraying with orcinol-H_2_SO_4_ reagent [18] followed by heating at 110 °C. To develop isolated GSLs at high resolution, silica gel 60 HPTLC plates (Merck KGaA, Germany) were used after separation on an Iatrobeads column.

### Gas chromatography

GC analyses of GSL components, such as sugars, fatty acids, and sphingoids, and methylation procedures were performed as described previously [9]. For the GC analysis, the following amount of GSL was used: 0.2 mg for sugar component analysis, fatty acid analysis, and methylation study, and 0.3 mg for sphingoid analysis.

### Matrix-assisted laser desorption/ionization time-of-flight mass spectrometry (MALDI-TOF MS)

MALDI-TOF MS analysis was performed using a Shimadzu Axima Confidence MALDI Mass Spectrometer with a nitrogen laser (337 nm). The matrix α-cyano-4-hydroxycinnamic acid (CHCA: high-purity mass-spectrometric grade) was purchased from Shimadzu GLC (Tokyo, Japan). About 4 μg of GSL was loaded on to a MALDI plate and air-dried. Subsequently, 1 μL of CHCA dissolved in 50 % ethanol at a saturating concentration was loaded 4 times on the dried GSL. External mass calibration was provided by the [M+Na]^+^ ions of angiotensin I (1296.96 mass units; Sigma-Aldrich Co., USA) and bradykinin fragments I–V (573.31 mass units; Sigma-Aldrich Co., USA).

### Proton nuclear magnetic resonance spectroscopy (^1^H-NMR spectroscopy)

Anomeric configurations were confirmed by ^1^H-NMR as previously reported [9].

## Results

### Purified neutral glycosphingolipids

**Fig. 1:**
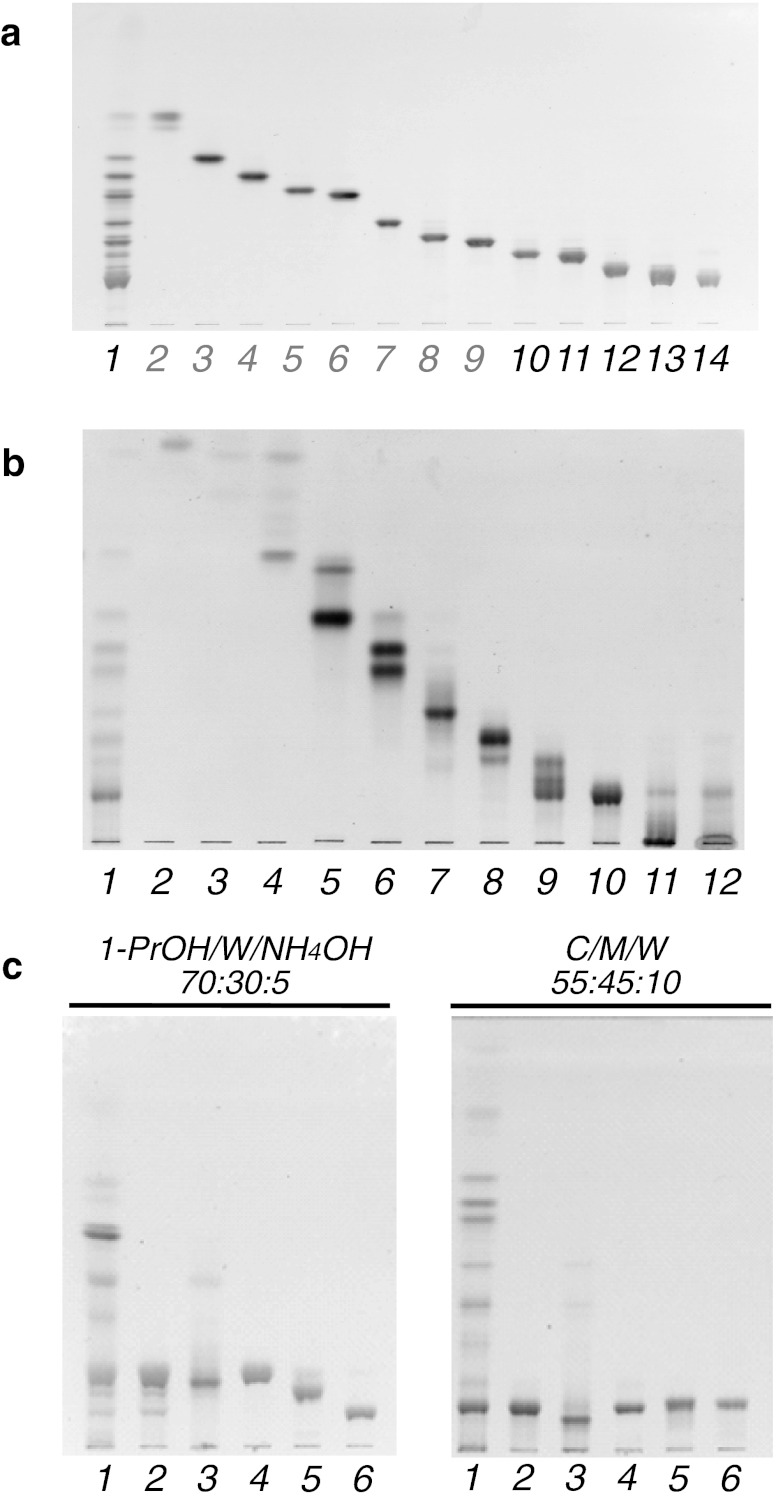
A thin-layer chromatogram shows the separation of neutral glycosphingolipids separated from the brine shrimp *A. franciscana*. **a** Fractionation by linear gradient elution. Lane 1, total neutral glycosphingolipid fraction; lanes 2–9, previously reported CMS, CDS, nAtCTS, AtCTS, nAtCTeS, AtCTeS, CPS, and CHS, respectively; lane 10, CHpS_1_; lane 11, CHpS_2_; lane 12, COS_1_; lane 13, mixture of COS_2_, CNS, and CDeS; lane 14, CDeS. **b** Fractionation by stepwise elution. Lane 1, total neutral glycosphingolipid fraction; lanes 2 and 3, nonpolar material fraction; lane 4, CMS; lane 5, CMS and CDS; lane 6, nAtCTS, AtCTS, and nAtCTeS; lane 7, AtCTeS; lane 8, CPS and CHS; lane 9, CHpS_1_, CHpS_2_, and COS_1_; lane 10, mixture of COS_2_, CNS, and CDeS; lanes 11 and 12, non-GSL fractions. **c** Purification of the fraction containing COS_2_, CNS, and CDeS by Iatrobeads column chromatography using ammoniacal propanol. Lane 1, total neutral glycosphingolipid fraction; lane 2, before fractionation; lane 3, non-GSL fraction; lane 4, CDeS; lane 5, CNS; lane 6, COS_2_. The HPTLC (**a** and **c**) and TLC (**b**) plates were developed in (**a** and **b**) C/M/W (60:40:10, v/v/v), (**c**) 1-propanol/water/ammonium hydroxide (70:30:5, v/v/v) or C/M/W (55:45:10, v/v/v). The spots were visualized by orcinol- H_2_SO_4_ reagent

Figure 1 shows a developed TLC plate with separated GSLs. The GSLs with less than six sugar residues, corresponding to lanes 2–9 in Fig. 1a, have been previously reported [9]. The yields of purified GSLs obtained from 1.8 kg of brine shrimp cysts were 1.0 mg (CHpS_1_), 1.3 mg (CHpS_2_), 1.3 mg (COS_1_), and 17.7 mg (CDeS). COS_2_ and CNS were eluted almost simultaneously and with relatively small yields. Therefore, to separate these two lipids, the entire procedure from lipid extraction to purification was repeated with an additional purification step capable of separating COS_2_ and CNS. The yield of Iatrobeads column chromatography after stepwise elution was as follows: ∼1 mg (lane 2), 5.6 mg (lane 3), 18.6 mg (lane 4), 2.6 mg (lane 5), 10.7 mg (lane 6), 5.5 mg (lane 7), 8.8 mg (lane 8), 8.1 mg (lane 9), 19.3 mg (lane 10), 1.5 mg (lane 11), and 3.3 mg (lane 12) (Fig. 1b). Crude COS_2_ (1.4 mg) and CNS (∼1 mg) were separated with an eluent of ammoniacal propanol. However, it was revealed in subsequent MALDI-TOF MS spectra that both COS_2_ and CNS preparations contained silicic acid. Further purification using a Sep-pak C18 cartridge was performed and the resulting lipids were again analyzed by MALDI-TOF MS. The non-GSL fraction was analyzed on the basis of the mass spectrum.

### Sugar composition

Each GSL was revealed to be an *N*-acetyl-*O*-trimethylsilyl derivatized methylglycoside by GC analysis. Sugar compositions were speculated according to their GCs: CHpS_1_ and CHpS_2_ yielded Glc, Man, Fuc, GlcNAc, and GalNAc in a molar ratio of 1:1:1:3:1; COS_1_ yielded Glc, Man, Fuc, GlcNAc, and GalNAc in a molar ratio of 1:1:1:3:2; and CDeS yielded Glc, Man, Fuc, GlcNAc, and GalNAc in a molar ratio of 1:1:2:4:2 (see Table 1).

**Table 1:** Molar ratios of sugar compositions in the complex neutral glycosphingolipids purified from the brine shrimp *A. franciscana*. The methylglycoside TMS derivatives of each GSL were analyzed by GC, and the results were expressed relative to Glc (= 1.0)

	Glc	Man	Fuc	GlcNAc	GalNAc
CHpS_1_	1.0	1.0	0.8	3.2	1.5
CHpS_2_	1.0	1.0	0.9	3.5	1.8
COS_1_	1.0	0.9	0.9	3.5	2.4
CDeS	1.0	0.9	1.6	3.9	2.5

### Methylation analysis

**Fig. 2:**
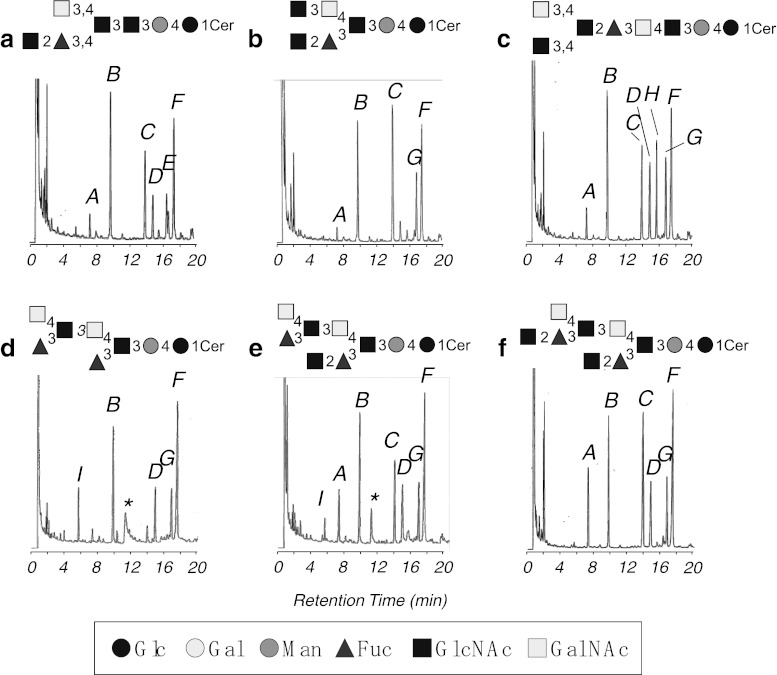
Gas chromatograms of partially methylated alditol acetates derived from the separated GSLs. (**a**) CHpS_1_; (**b**) CHpS_2_; (**c**) COS_1_; (**d**) COS_2_; (**e**) CNS; (**f**) CDeS; *A*, 1,2Fuc; *B*, 1,4Glc and 1,3Man; *C*, 1GlcNAc; *D*, 1GalNAc; *E*, 1,3GlcNAc; *F*, 1,3,4GlcNAc; *G*, 1,3GalNAc; *H*, 1,4GlcNAc; *I*, 1Fuc, *, contaminant of phthalic acid-like material

The partially methylated alditol acetates derived from the obtained GSLs were separated by GC, as shown in Fig. 2. The methylation analysis revealed 1,2,5-tri-*O*-acetyl-3,4-di-*O*-methylfucitol (1,2Fuc), 1,4,5-tri-*O*-acetyl-2,3,6-tri-*O*-methylglucitol (1,4Glc), 1,3,5-tri-*O*-acetyl-2,4,6-tri-*O*-methylmannitol (1,3Man), 1,5-di-*O*-acetyl-3,4,6-tri-*O*-methyl-*N*-acetylglucosaminitol (1GlcNAc), 1,5-di-*O*-acetyl-3,4,6-tri-*O*-methyl-*N*-acetylgalactosaminitol (1GalNAc), 1,3,5-tri-*O*-acetyl-4,6-di-*O*-methyl-*N*-acetylglucosaminitol (1,3GlcNAc), and 1,3,4,5-tetra-*O*-acetyl-6-*O*-methyl-*N*-acetylglucosaminitol (1,3,4GlcNAc) from CHpS_1_; 1,4Glc, 1,3Man, 1,2Fuc, 1GlcNAc, 1,3,5-tri-*O*-acetyl-4,6-di-*O*-methyl-*N*-acetylgalactosaminitol (1,3GalNAc), and 1,3,4GlcNAc from CHpS_2_; 1,2Fuc, 1,4Glc, 1,3Man, 1GlcNAc, 1GalNAc, 1,4,5-tri-*O*-acetyl-3,6-di-*O*-methyl-*N*-acetylglucosaminitol (1,4GlcNAc), 1,3GalNAc, and 1,3,4GlcNAc from COS_1_; 1,5-di-*O*-acetyl-2,3,4-tri-*O*-methylfucitol (1Fuc), 1,4Glc, 1,3Man, 1GalNAc, 1,3GalNAc, and 1,3,4GlcNAc from COS_2_; 1Fuc, 1,2Fuc, 1,4Glc, 1,3Man, 1GlcNAc, 1GalNAc, 1,3GalNAc, and 1,3,4GlcNAc from CNS; 1,2Fuc, 1,4Glc, 1,3Man, 1GlcNAc, 1GalNAc, 1,3GalNAc, and 1,3,4GlcNAc from CDeS. 1,4Glc and 1,3Man were eluted at the same retention time and observed as a high peak B. Although methylation analysis of CHpS_1_ showed the presence of 1,3GalNAc, CHpS_1_ also contained a small amount of CHpS_2_ as demonstrated by HPTLC analysis using an ammoniacal solvent (Data not shown).

### Aliphatic components

Aliphatic components such as fatty acids and sphingoids were identified by GC (Table 2). The majority of the fatty acids were saturated with chain lengths from C_16_ to C_24_, with C_22_ being the most predominant. The monoenoic acid of C_22_ was common in all of the GSLs. In some GSLs, the odd-numbered saturated fatty acids C_21_ and C_23_ were also detected in low amounts. The sphingoid components of the GSLs were composed of d16:1 and d17:1. In each case, the amount of d16:1 was approximately double that of d17:1.

**Table 2:** Ceramide composition of the neutral glycosphingolipids purified from *A. franciscana*

Fatty acid (%)	CHpS_1_	CHpS_2_	COS_1_	CDeS
16:1	–^b^	–	–	tr ^c^
16:0	1.0	tr	1.1	4.1
18:1	–	–	–	3.4
18:0	3.7	5.6	4.8	5.8
20:0	1.5	2.0	1.9	2.1
21:0	tr	1.1	1.0	1.0
22:1	4.2	3.8	3.8	1.1
22:0	84.3	84.4	82.2	79.4
23:0	1.4	1.1	1.3	1.1
24:1	1.0	tr	1.4	–
24:0	2.9	2.0	2.5	2.0

Sphingoid (%)				
d16:1^a^	66.7	69.3	65.7	62.3
d17:1	33.3	30.7	34.3	37.7

^a^Dihydroxy sphingoid

^b^Not detected

^c^Trace

### MALDI-TOF MS analysis

**Fig. 3:**
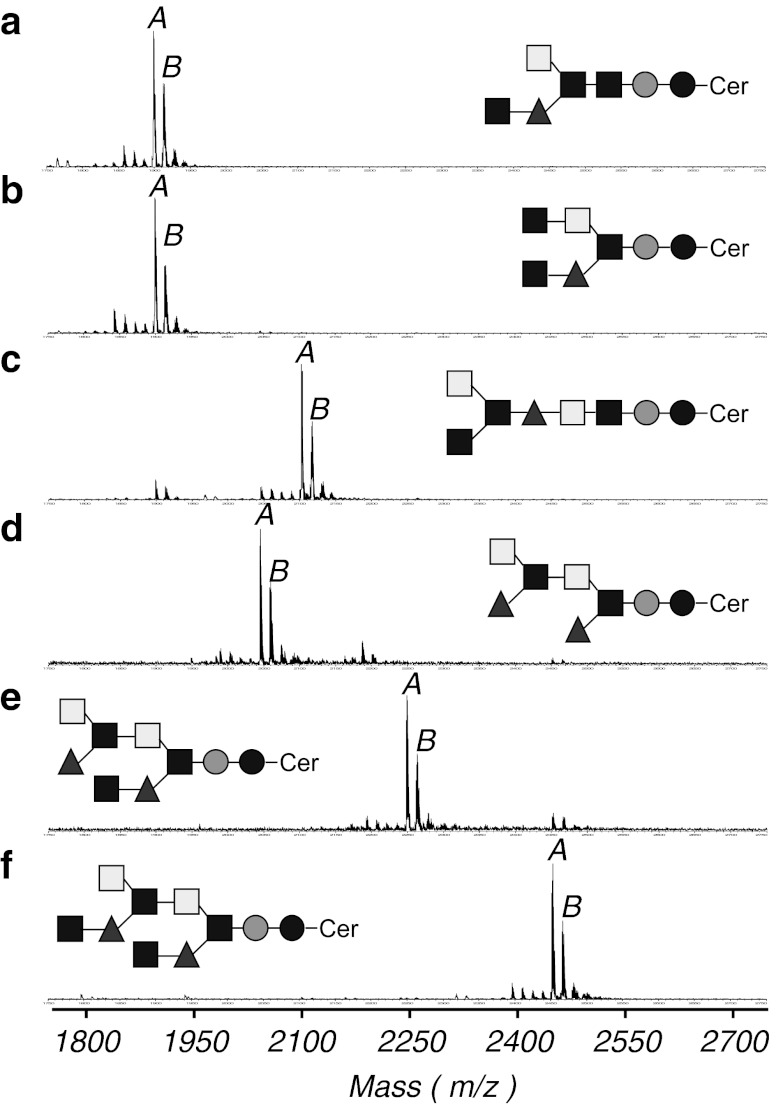
Positive-ion reflector mode MALDI-TOF MS spectra of the separated GSLs. **a** CHpS_1_; *A*, [M+Na]^+^ ion at *m/z* 1899.47; *B*, [M+Na]^+^ ion at *m/z* 1913.42; **b** CHpS_2_; *A*, [M+Na]^+^ ion at *m/z* 1898.82; *B*, [M+Na]^+^ ion at *m/z* 1912.78; **c** COS_1_; *A*, [M+Na]^+^ ion at *m/z* 2101.84; *B*, [M+Na]^+^ ion at *m/z* 2115.81; **d** COS_2_; *A*, [M+Na]^+^ ion at *m/z* 2044.96; *B*, [M+Na]^+^ ion at *m/z* 2058.95; **e** CNS; *A*, [M+Na]^+^ ion at *m/z* 2248.38; *B*, [M+Na]^+^ ion at *m/z* 2262.31; **f** CDeS; *A*, [M+Na]^+^ ion at *m/z* 2451.45; *B*, [M+Na]^+^ ion at *m/z* 2465.42

The positive in-source decay mode of MALDI-TOF MS confirmed the putative structures of the six purified GSLs (Fig. 3). Each GSL mass spectrum contained peaks attributed to two major monoisotopic [M+Na]^+^ ion species, the ceramide moieties of d16:1–22:0 and d17:1–22:0. For CHpS_1_, these peaks correspond to ions at *m/z* 1899.47 and 1913.42 with one mole each of Glc, Man, GalNAc, and Fuc and three moles of GlcNAc (Fig. 3a). For CHpS_2_, the peaks correspond to ions at *m/z* 1898.82 and 1912.78 with one mole each of Glc, Man, GalNAc, and Fuc and three moles of GlcNAc (Fig. 3b). For COS_1_, ion peaks at *m/z* 2101.84 and 2115.81 indicated one mole each of Glc, Man, and Fuc, two moles of GalNAc, and three moles of GlcNAc (Fig. 3c). For COS_2_, the ion peaks at *m/z* 2044.96 and 2058.95 corresponded to one mole each of Glc, Man, two moles each of Fuc, GalNAc, and GlcNAc and three moles of GlcNAc (Fig. 3d). For CNS, the ion peaks at *m/z* 2248.38 and 2262.31 corresponded to one mole each of Glc and Man, two moles of GalNAc and Fuc, and three moles of GlcNAc (Fig. 3e). For CDeS, the ion peaks at *m/z* 2451.45 and 2465.42 corresponded to one mole each of Glc and Man, two moles of GalNAc and Fuc, and four moles of GlcNAc (Fig. 3f). Detailed masses of ions other than those corresponding to the major [M+Na]^+^ ion species are summarized in Supplemental table.

**Fig. 4:**
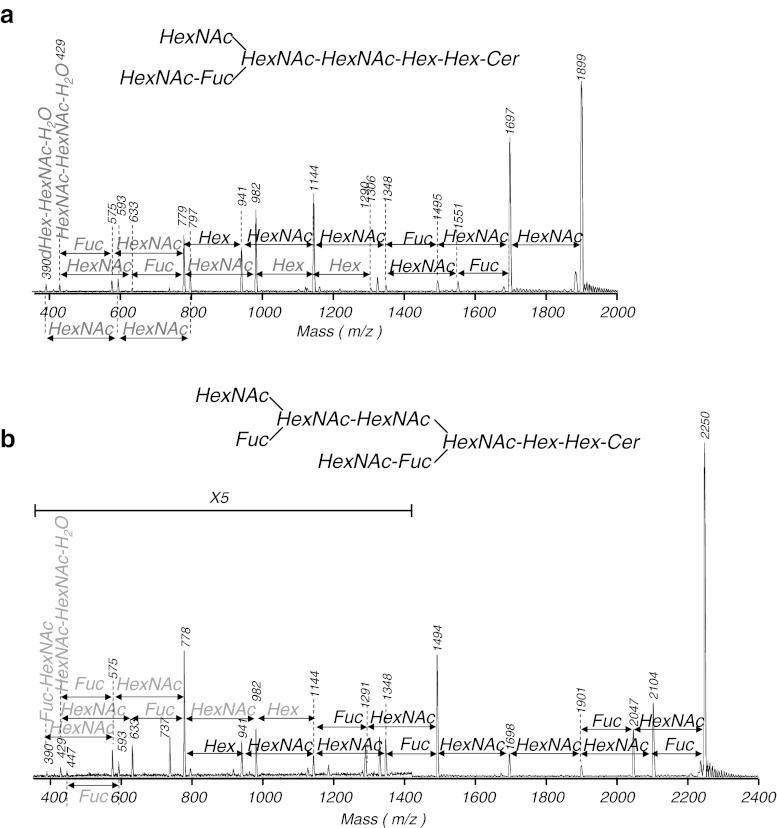
Representative positive-ion PSD spectra in MALDI-TOF MS of the separated GSLs. **a** CHpS_1_; **b** CNS. *Black arrows* indicate mass differences between fragments with a ceramide molecular group. *Gray arrows* indicate mass differences between fragments without a ceramide molecular group. All fragments were detected as sodium adducts. The spectrum of **b** was expanded by a factor of 5 in the low-molecular-weight region

The sugar sequence of each GSL was determined by MALDI-TOF PSD measurements, as shown in Fig. 4. The predominant [M+Na]^+^ ion was chosen as the precursor ion in MALDI-PSD fragment spectra. All fragment ions were observed as sodium adducts. In the PSD mass spectrum of CHpS_1_, fragment ions showing a neutral loss of sugar were observed at *m/z* 1697 ([M−HexNAc+Na]^+^), 1551 ([M−HexNAc−Fuc+Na]^+^), 1495 ([M−2HexNAc+Na]^+^), 1348 ([M−2HexNAc−Fuc+Na]^+^), 1144 ([M−3HexNAc−Fuc+Na]^+^), 941 ([M−4HexNAc−Fuc+Na]^+^), and 779 ([M−4HexNAc−Fuc−Hex+Na]^+^), corresponding to CMS with d16:1/C22:0 (Fig. 4a). In the PSD mass spectrum of CHpS_2_, fragment ions showing a neutral loss of sugar were observed at *m/z* 1699 ([M−HexNAc+Na]^+^), 1551 ([M−HexNAc−Fuc+Na]^+^), 1496 ([M−2HexNAc+Na]^+^), 1349 ([M−2HexNAc−Fuc+Na]^+^), 1292 ([M−3HexNAc+Na]^+^), 1145 ([M−3HexNAc−Fuc+Na]^+^), 942 ([M−4HexNAc−Fuc+Na]^+^), and 779 ([M−4HexNAc−Fuc−Hex+Na]^+^), corresponding to CMS with d16:1/C22:0 (Supplemental Fig. 1a). In the PSD mass spectrum of COS_1_, fragment ions showing a neutral loss of sugar were observed at *m/z* 1901 ([M−HexNAc+Na]^+^), 1697 ([M−2HexNAc+Na]^+^), 1494 ([M−3HexNAc+Na]^+^), 1348 ([M−3HexNAc−Fuc+Na]^+^), 1145 ([M−4HexNAc−Fuc+Na]^+^), 941 ([M−5HexNAc−Fuc+Na]^+^), and 779 ([M−5HexNAc−Fuc−Hex+Na]^+^), corresponding to CMS with d16:1/C22:0 (Supplemental Fig. 1b). In the PSD mass spectrum of COS_2_, fragment ions showing a neutral loss of sugar were observed at *m/z* 1901 ([M−Fuc+Na]^+^), 1843 ([M−HexNAc+Na]^+^), 1754 ([M−2Fuc+Na] ^+^), 1697 ([M−HexNAc−Fuc+Na]^+^), 1552 ([M−HexNAc−2Fuc+Na]^+^), 1494 ([M−2HexNAc−Fuc+Na]^+^), 1348 ([M−2HexNAc−2Fuc+Na]^+^), 1290 ([M−3HexNAc−Fuc+Na]^+^), 1145 ([M−3HexNAc−2Fuc+Na]^+^), 941 ([M−4HexNAc−2Fuc+Na]^+^), and 779 ([M−4HexNAc−2Fuc−Hex+Na]^+^), corresponding to CMS with d16:1/C22:0 (Supplemental Fig. 1c). In the PSD mass spectrum of CNS, fragment ions showing a neutral loss of sugar were observed at *m/z* 2104 ([M−Fuc+Na]^+^), 2047 ([M−HexNAc+Na]^+^), 1901 ([M−HexNAc−Fuc+Na]^+^), 1698 ([M−2HexNAc−Fuc+Na]^+^), 1494 ([M−3HexNAc−Fuc+Na]^+^), 1348 ([M−3HexNAc−2Fuc+Na]^+^), 1291 ([M−4HexNAc−Fuc+Na]^+^), 1144 ([M−4HexNAc−2Fuc+Na]^+^), 941 ([M−5HexNAc−2Fuc+Na]^+^), and 779 ([M−5HexNAc−2Fuc−Hex+Na]^+^), corresponding to CMS with d16:1/C22:0 (Fig. 4b). In the PSD mass spectrum of CDeS, fragment ions showing a neutral loss of sugar were observed at *m/z* 2250 ([M−HexNAc+Na]^+^), 2104 ([M−HexNAc−Fuc+Na]^+^), 2046 ([M−2HexNAc+Na]^+^), 1900 ([M−2HexNAc−Fuc+Na]^+^), 1697 ([M−3HexNAc−Fuc+Na]^+^), 1494 ([M−4HexNAc−Fuc+Na]^+^), 1348 ([M−4HexNAc−2Fuc+Na]^+^), 1290 ([M−5HexNAc−Fuc+Na]^+^), 1144 ([M−5HexNAc−2Fuc+Na]^+^), 940 ([M−6HexNAc−2Fuc+Na]^+^), and 778 ([M−6HexNAc−2Fuc−Hex+Na]^+^), corresponding to CMS with d16:1/C22:0 (Supplemental Fig. 1d).

### Anomeric configurations of the sugar residues

**Fig. 5:**
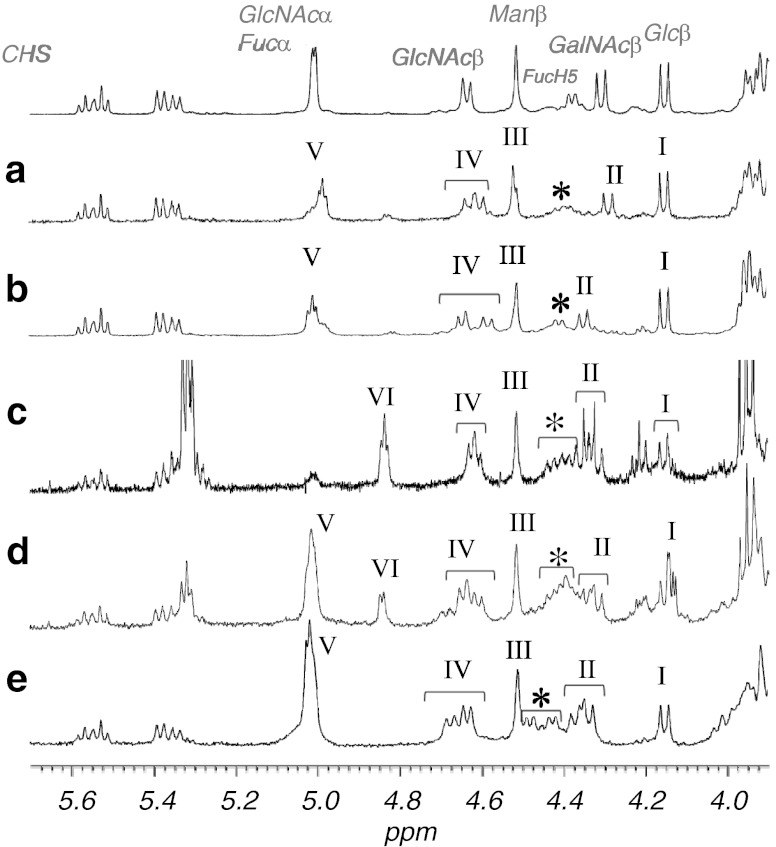
Anomeric proton regions of the ^1^H-NMR spectra of the separated GSLs. **a** CHpS_1_; **b** CHpS_2_; **c** COS_2_; **d** CNS; **e** CDeS; I: Glcβ; II: GalNAcβ; III: Manβ; IV: GlcNAcβ; V: Fucα and GlcNAcα; VI: Fucα; *: FucH5

Anomeric protons of the sugar residues in CHpSes and CDeS were analyzed by ^1^H-NMR spectroscopy (Fig. 5 and Table 3). The configurations of these residues were assigned by comparisons with similar data obtained on MacCer and At_3_Cer [19], At_5_Cer [20], Fuc [21], and α-GlcNAc [22, 23] (Table 3). The anomeric assignments of α-Fuc and α-GlcNAc were determined by a downfield shift of the glycosyl-substituted fucose peak relative to that in the NMR spectrum of non-arthro-CTS [9], which was consistent with the data of Xu *et al.* [24]. Anomeric proton resonances are shown in Fig. 5 for each of the GSLs. The glycoside linkages in these compounds were also speculated. In the anomeric region of the spectrum for each GSL, the anomeric proton resonances are observed at 4.16 ppm (*J*_1,2_ = 7.8 Hz) for β-Glc, at 4.52 ppm (*J*_1,2_ = ∼1 Hz) for β-Man, at 4.60 and 4.63 ppm (*J*_1,2_ = 8.2 and 9.1 Hz, respectively) for β-GlcNAc, at 4.29 ppm (*J*_1,2_ = 8.2 Hz) for β-GalNAc, and at 4.99 and 4.98 ppm (*J*_1,2_ = 3.2 and 2.7 Hz) for overlapped α-Fuc and α-GlcNAc (Fig. 5a, CHpS_1_); at 4.15 ppm (*J*_1,2_ = 7.8 Hz) for β-Glc, at 4.51 ppm (*J*_1,2_ = ∼1 Hz) for β-Man, at 4.58 and 4.65 ppm (*J*_1,2_ = 7.3 and 7.8 Hz, respectively) for β-GlcNAc, at 4.35 ppm (*J*_1,2_ = 7.8 Hz) for β-GalNAc, and at 5.01 and 5.02 ppm (*J*_1,2_ = 3.4 and 3.6 Hz) for overlapped α-Fuc and α-GlcNAc (Fig. 5b, CHpS_2_); at 4.15 or 4.16 ppm (*J*_1,2_ = 6.4 or 8.7 Hz) for β-Glc, at 4.52 ppm (*J*_1,2_ = ∼1 Hz) for β-Man, at 4.63 and 4.64 ppm (*J*_1,2_ = 7.3 Hz) for β-GlcNAc, at 4.32 and 4.34 ppm (*J*_1,2_ = 6.9 and 7.8 Hz, respectively) for β-GalNAc, at 5.01 ppm (*J*_1,2_ = 2.7 Hz) for overlapped α-Fuc and α-GlcNAc, and 4.84 ppm (*J*_1,2_ =3.7 Hz) for terminal α-Fuc (Fig. 5d, CNS); at 4.15 ppm (*J*_1,2_ = 7.6 Hz) for β-Glc, at 4.51 ppm (*J*_1,2_ = ∼1 Hz) for β-Man, at 4.64 and 4.68 ppm (*J*_1,2_ = 7.2 and 7.4 Hz, respectively) for β-GlcNAc, at 4.34 and 4.37 ppm (*J*_1,2_ = 8.0 and 8.1 Hz, respectively) for β-GalNAc, and at 5.02 ppm (*J*_1,2_ = 2.9 Hz) for overlapped α-Fuc and α-GlcNAc (Fig. 5e, CDeS). As for COS_2_, purity was low that significant high signals attributable to a contaminant were observed at the olefin signal region. Although precise coupling constants could not be calculated, anomeric configuration and sugar species of COS_2_ were annotated according to chemical shift by comparing other NMR spectra of *Artemia* GSLs.

**Table 3:** Summary of chemical shifts and *J*_*1,2*_ coupling constants of anomeric protons in complex GSLs purified from *A. franciscana*

CHpS_1_				GalNAc1-3,4	(GlcNAc1-	2Fuc1-3,4)	GlcNAc1-	3GlcNAc1-	3Man1-	4Glc1
				II	V	V	IV	IV	III	I
Chemical shifts (ppm)				4.29	4.98	4.98	4.60	4.60	4.52	4.16
					4.99	4.99	4.63	4.63		
Coupling constants (Hz)				8.2	2.7	2.7	8.2	8.2	∼1	7.8
					3.2	3.2	9.1	9.1		

CHpS_2_				GlcNAc1-3	GalNAc1-4	(GlcNAc1-	2Fuc1-3)	GlcNAc1-	3Man1-	4Glc1
				IV	II	V	V	IV	III	I
Chemical shifts (ppm)				4.58	4.35	5.01	5.01	4.58	4.51	4.15
				4.65		5.02	5.02	4.65		
Coupling constants (Hz)				7.3	7.8	3.4	3.4	7.3	∼1	7.8
				7.8		3.6	3.6	7.8		

CNS		GalNAc1-4	(Fuc1-3)	GlcNAc1-3	GalNAc1-4	(GlcNAc1-	2Fuc1-3)	GlcNAc1-	3Man1-	4Glc1
		II	VI	IV	II	V	V	IV	III	I
Chemical shifts (ppm)		4.32	4.84	4.63	4.32	5.01	5.01	4.63	4.52	4.15
		4.34		4.64	4.34			4.64		4.16
Coupling constants (Hz)		6.9	3.7	7.3	6.9	2.7	2.7	7.3	∼1	6.4
		7.8		7.3	7.8			7.3		8.7

CDeS	GalNAc1-4	(GlcNAc1-	2Fuc1-3)	GlcNAc1-3	GalNAc1-4	(GlcNAc1-	2Fuc1-3)	GlcNAc1-	3Man1-	4Glc1
	II	V	V	IV	II	V	V	IV	III	I
Chemical shifts (ppm)	4.34	5.02	5.02	4.64	4.34	5.02	5.02	4.64	4.51	4.15
	4.37			4.68	4.37			4.68		
Coupling constants (Hz)	8.0	2.9	2.9	7.2	8.0	2.9	2.9	7.2	∼1	7.6
	8.1			7.4	8.1			7.4		

### Speculated structures of the complex GSLs

The following complex GSL structures were speculated by means of the conventional analyses plus MALDI PSD measurements: IV^3^-(GlcNAcβ)-III^3^-(GlcNAcα2Fucα)-At_4_Cer (CHpS_2_), IV^3^-(fucosyl-LacdiNAc)-III^3^-(GlcNAcα2Fucα)-At_4_Cer (CNS), and IV^3^-(2-α-*N*-acetylglucosaminylfucosyl-LacdiNAc)-III^3^-(GlcNAcα2Fucα)-At_4_Cer (CDeS). The following complex GSL structures were also analyzed: IV^3,4^-(GalNAcβ)-IV^3,4^-(GlcNAcα2Fucα)-At_4_Cer (CHpS_1_), GalNAc1-3,4(GlcNAc1-3,4)GlcNAc1-2Fuc1-3GalNAc1-4GlcNAc1-3Man1-4Glc1-Cer (COS_1_), and IV^3^- (fucosyl-LacdiNAc)-III^3^-(Fucα)-At_4_Cer (COS_2_). Although “1,3,4GlcNAc” was observed as a branching GlcNAc in both CHpS_1_ and COS_1_, it was impossible to confirm sugar substitution at the C3/C4 position without hydrolysis analysis, which requires a large amount of CHpS_1_ and COS_1_.

## Discussion

Structural characterization of complex GSLs can be a painstaking process because the results obtained by different analyses must converge on a single structure. For example, characterization by liquid chromatography-mass spectrometry (LC-MS) and tandem MS (MS-MS) provides accurate mass values of ceramide moieties, fatty acids, and sphingoids, but not of sugar species. It is also possible that liquid chromatographic separation would not be able to fully separate GSLs with different sugar chains of identical molecular weight. The conventional analyses, which consist of GC and MS, can be used to determine sugar species and molar ratios without overlooking the possibility of GSLs with different sugar chains of identical molecular weight. Another benefit of the conventional analysis is the ability to determine branching structures by hydrolysis. No matter the analysis, it is difficult to characterize complex GSLs because each result is also complex. A few decades ago, characterization of a complex GSL required 10 mg of isolated GLS for chemical degradation, which liberated shorter GSLs, followed by GC analysis to determine sugar sequence and exoglycosidase treatment or chromic acid oxidation to determine anomeric configurations. In this study, we conducted MALDI PSD analysis to determine sugar sequence and ^1^H-NMR spectroscopy to identify anomeric configurations by using only 1-mg samples of each complex GSL separated from *A. franciscana*. Although PSD spectra of complex GSLs were confusing, sugar sequence analysis using sequences speculated from fragments, with the ceramide moiety observed in the high mass region, were consistent with sequences speculated from mere glycan fragments observed in the low mass region.

This study examined the structures of complex GSLs separated from the brine shrimp *A. franciscana*. Of them, a fucosylated LacdiNAc trisaccharide structure (GalNAcβ1-4[Fucα1-3]GlcNAcβ) was found in CHpS_2_, COS_2_, CNS, and CDeS, similar to a previous report on CHS [9]. The trisaccharide structure is an analog of Lewis X and is also found in GSLs from the parasite *S. mansoni* [25] and in the carbohydrate portion of a human immunosuppressive glycoprotein, glycodelin [26].

The arthro-series core structure and a branching non-arthro-series disaccharide were also found in CHpS_2_, CNS, and CDeS, as described in previous reports on nAtCTeS and CHS. The α-anomeric GlcNAc residue is rare, and there have been few reports of this residue among the carbohydrate portions of GSLs and glycoproteins. A human gastric mucin with an α-anomeric GlcNAc residue could arrest the proliferation of *Helicobacter pylori* [27].

**Fig. 6:**
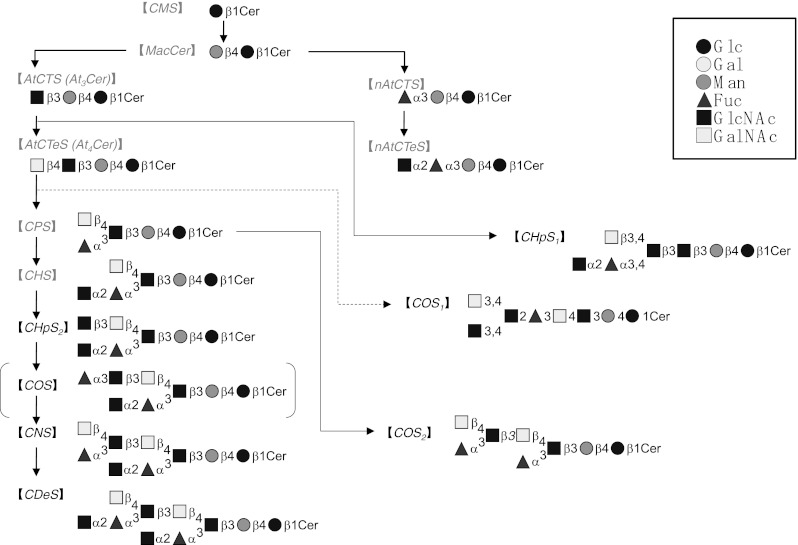
Summary of the putative biosynthetic pathway of non-arthro and arthro-series neutral GSLs in the cyst of the brine shrimp

We speculate that CHpS_2_ is elongated with an additional Fucα group, forming COS, and that COS is further elongated with branching GalNAcβ to form CNS. Although we could not separate COS as a direct precursor of CNS, we believe it exists in the biosynthetic pathway (Fig. 6). CNS is then finally elongated with an additional GlcNAcα to form CDeS. However, another branching residue on CHpS_1_ and COS_1_ indicates other biosynthetic pathways. *A. franciscana* GSLs with α-anomeric GlcNAc at the nonreducing end do not seem to be elongated with further saccharides, which suggests the action of a “capping” mechanism. This theory is further supported by the observation that all of the α-anomeric GlcNAc residues were reported at the nonreducing end [9, 22–24, 28, 29]. Capping by an amino sugar in *A. franciscana* GSLs may be similar to the capping action of the 2-keto-3-deoxy-d-*glycero*-d-*galacto*-nononic acid (KDN) residue in the rainbow trout *Salmo gairdneri* [30].

A series of structural analyses, including this study, has established the existence of sphingomyelin as a sphingophospholipid and GlcCer, MacCer, At_3_Cer, II^3^Fucα-MacCer (non-arthro-CTS), At_4_Cer, II^3^(GlcNAcα2Fucα)-MacCer (non-arthro-CTeS), III^3^Fucα-At_4_Cer (CPS), III^3^(GlcNAcα2Fucα)-At_4_Cer (CHS), CHpS_1_, CHpS_2_, COS_1_, COS_2_, CNS, and CDeS as GSLs of *A. franciscana*. The complex GSLs reported this time are as the novel fucomannolipids. However, we confirmed that gangliosides and their functional alternative materials (other acidic GSLs) were below the limit of detection. In mammals, acidic GSLs such as gangliosides play important roles in the formation of the plasma membrane surface environment for signal transduction. It is interesting that *A. franciscana* can survive without significant amounts of acidic GSLs.

Performing structural analyses of complex GSLs in *A. franciscana* cysts, which are developmentally diapaused gastrulae, we established a GSL profile during embryonic development. Similar GSL analyses have been carried out in other species, including the frog *Xenopus laevis* [31], the chicken *Gallus gallus domesticus* [32], and the mouse *Mus musculus* [33]. However, all of these studies were conducted during the neurula stage or later, emphasizing the importance of timing in our study. *A. franciscana* is a very suitable organism for studying GSL profiles during early embryogenesis, as they require 5 days from fertilization to the gastrula stage.

In the brine shrimp *A. franciscana*, there are two reproduction patterns: oviparity, which generates diapausing eggs (cysts), and ovoviviparity, which generates nauplii. Oviparous eggs are resistant to dryness due to the existence of the heat shock protein p26 [34], which seems to be induced by environmental factors than by temperature. Nambu *et al.* [35] conducted experiments showing that certain conditions of light/dark cycles and temperature could affect the reproduction pattern of brine shrimp. GSLs have been shown to be involved in signaling, and signal transduction is, at least in part, responsible for the choice of oviparity or ovoviviparity. Therefore, this study, which provides a comprehensive analysis of GSLs in embryonic *A. franciscana*, might be of major significance in studies of embryogenesis and signal transduction.

*A. franciscana* contains complex GSLs such as CDeS, CHpSes, and COSes. Other invertebrates containing complex GSLs with more than five sugar residues are [5] the flies *L. caesar*, *C. vicina*, *Drosophila melanogaster* (Insecta), the parasitic nematode *Ascaris suum* (Nematoda), the liver fluke *Fasciola hepatica*, the blood fluke *S. mansoni* (Platyhelminthes: Trematoda), the pseudophyllidean tapeworm *Spirometra erinacei*, the tapeworm *Diphyllobothrium hottai* (Platyhelminthes: Cestoda), the earthworm *Pheretima hilgendorfi* (Annelida: Oligochaeta), the annelidan worm *Pseudopotamilla occelata* (Annelida: Polychaeta), the bivalves *H. schlegeli*, *C. sandai*, *M. lusoria*, the oyster *O. gigas* (Mollusca), the abalone *Haliotis japonica* (Mollusca: Gatropoda), the lamp shell *Lingula unguis* (Brachiopoda), and the sea urchin *Hemicentrotus pulcherrimus* (Echinodermata). Of these, only half contain GSLs with the same number of sugar residues and a different sugar chain sequence. As in vertebrates, GSLs with multiple sugar chain sequences may exhibit tissue specificity. These GSLs can be roughly divided into two groups: one with straight complex GSLs, such as those found in flies, the nematode, the blood fluke, and the lamp shell, and those with branching complex GSLs. Complex GSLs with more than seven sugar residues have been reported as follows: GlcNAcβ1-3Galβ1-3GalNAcα1-4GalNAcβ1-4GlcNAcβ1-3Manβ1-4Glcβ1-Cer (At_7_Cer) from the fly *C. vicina* [11]; At_7_Cer, VII^3^-β-GalNAc-At_7_Cer (At_8_Cer), and VIII^3^-β-Gal-At_8_Cer (At_9_Cer) from the fly *L. caesar* [10]; Fucα3GalNAcβ4(Fucα3)GlcNAcβ3GlcNAcβ3GalNAcβ4GlcβCer, Fucα3GalNAcβ4(Fucα2Fucα3)GlcNAcβ3GlcNAcβ3GalNAcβ4GlcβCer, Fucα3GalNAcβ4(Fucα2Fucα2Fucα3)GlcNAcβ3GlcNAcβ3GalNAcβ4GlcβCer, and Fuc1-4(Fuc1-3)GlcNAc1-2Fuc1-4(Fuc1-3)GlcNAc1-2Fuc1-4(Fuc1-3)GlcNAc1-2Fuc1-4(Fuc1-3)GlcNAc1-3GalNAc1-3GalNAc1-4Glc1-Cer from the blood fluke [12–14]; Gal4Meβ1-3GalNAcβ1-3Fucα1-4GlcNAcβ1-2Manα1-3(Xylβ1-2)Manβ1-4Glcβ1-Cer (GL-1) from the bivalve *C. sandai* [15]; Fuc3Meα1-2Xyl3Meβ1-4(GalNAc3Meα1-3)Fucα1-4GlcNAcβ1-2Manα1-3(Xylβ1-2)Manβ1-4Glcβ1-Cer (Lipid III) from the bivalves *H. schlegeli* and *M. lusoria* [16, 17]; and GlcNAc1-3(Fuc3Meα1-2)Gal1-4GalNAc1-3[GalNAc3Me1-3(Fucα1-2)Gal1-2]Galβ1-3Galβ1-4Glcβ1-Cer from the oyster [5]. All of these GSLs, with the exception of those found in the flies, contain a branching sugar chain and at least one fucose residue. In addition, almost all of the molluscan GSLs contain xylose and an *O*-methyl sugar residue. Organisms can also be divided into two groups according to the presence or absence of acidic or polar GSLs. The former group includes the flies and the bivalves. The latter group contains *A. franciscana*, the blood fluke, and the oyster. Furthermore, a fucosylated LacdiNAc structure and a GlcNAcβ1-3GlcNAc disaccharide are common to both *A. franciscana* and the blood fluke. Shorter GSLs seem to be conserved within the same phyla while longer GSLs are more commonly unique to a given species.

This study of the entire GSL structure in *A. franciscana* showed that the ceramide composition is common among nearly all neutral GSLs, irrespective of the length of the sugar chain, with the exception of CMS, which contains hydroxy fatty acid, similar to millipedes [21], but not to flies such as *L. caesar* and *C. vicina* [6, 7, 10, 11, 36]. In arthropods, the dominant ceramide moieties are d16:1 and smaller amounts of d17:1 as sphingoid; C22:0 and smaller amounts of C18:0 as fatty acid in *A. franciscana*; d17:1 and smaller amounts of branched d18:1 as sphingoid; C22:0 and smaller amounts of C23:0/C24:0 as fatty acid in the millipede; d14:1 and smaller amounts of d16:1 as sphingoid; C20:0 and smaller amounts of C18:0/C22:0 as fatty acid in the flies and in the High Five insect cell line derived from the cabbage looper *Trichoplusia ni* [37]. As mentioned above, the ceramide composition is found in both *A. franciscana* and the millipede, especially odd-numbered sphingoids, with the highest amount being C22:0. In addition, all of the neutral GSLs isolated from flies were straight, while the millipede contained a branching neutral GSL [21] and a ceramide phosphoryl ethanolamine (CPEA) [38] instead of the polar GSL with a molecular group of phosphoryl-ethanolamine, as found in flies [5]. *A. franciscana* contained branching neutral GSLs and sphingomyelin instead of a sphingolipid with a phosphoryl-ethanolamine group [39]. This commonality might indicate that *A. franciscana* is more closely related to the millipede than to insects.

Currently, it is not possible to use molecular biological approaches in brine shrimp because its genome has not been sequenced. However, there has been a report detailing the RNAi knockdown of a homeotic gene using microinjection [40]. It has been speculated that glycosyltransferases could be found in the arthropodal genomic sequences of *D. melanogaster* and other species, which might aid in the elucidation of a relationship between morphogenesis and the functions of GSLs in Crustacea.

## Electronic supplementary material

Below is the link to the electronic supplementary material.

Fig. S1 Positive-ion PSD spectra in MALDI-TOF MS of the separated GSLs. (**a**) CHpS_2_; (**b**) COS_1_; (**c**) COS_2_; and (**d**) CDeS. (PDF 115 kb)

Table S1 Summary of MALDI-TOF MS spectra obtained for complex GSLs purified from *A. franciscana*. Symbols A and B, denoted in the left column, show predominant peaks mentioned in Fig. 3. % distribution was calculated using % value of aliphatic component analysis. tr: trace (PDF 49 kb)

## Abbreviations

CDeS: Ceramide decasaccharide
CHpS: Ceramide heptasaccharide
CHS: Ceramide hexasaccharide
CNS: Ceramide nonasaccharide
COS: Ceramide octasaccharide
GC: Gas chromatography
GSL: Glycosphingolipid
^1^H-NMR: Proton nuclear magnetic resonance
HPTLC: High-performance thin-layer chromatography
MALDI-TOF MS: Matrix-assisted laser desorption/ionization time-of-flight mass spectrometry
