# Supplementary figures and images for: Biochemical studies on sphingolipids of Artemia franciscana: complex neutral glycosphingolipids
Source: Glycoconj J. 2012 Aug 14;30(3):257–68. doi: 10.1007/s10719-012-9436-8 (PMC3606520; doi:10.1007/s10719-012-9436-8)

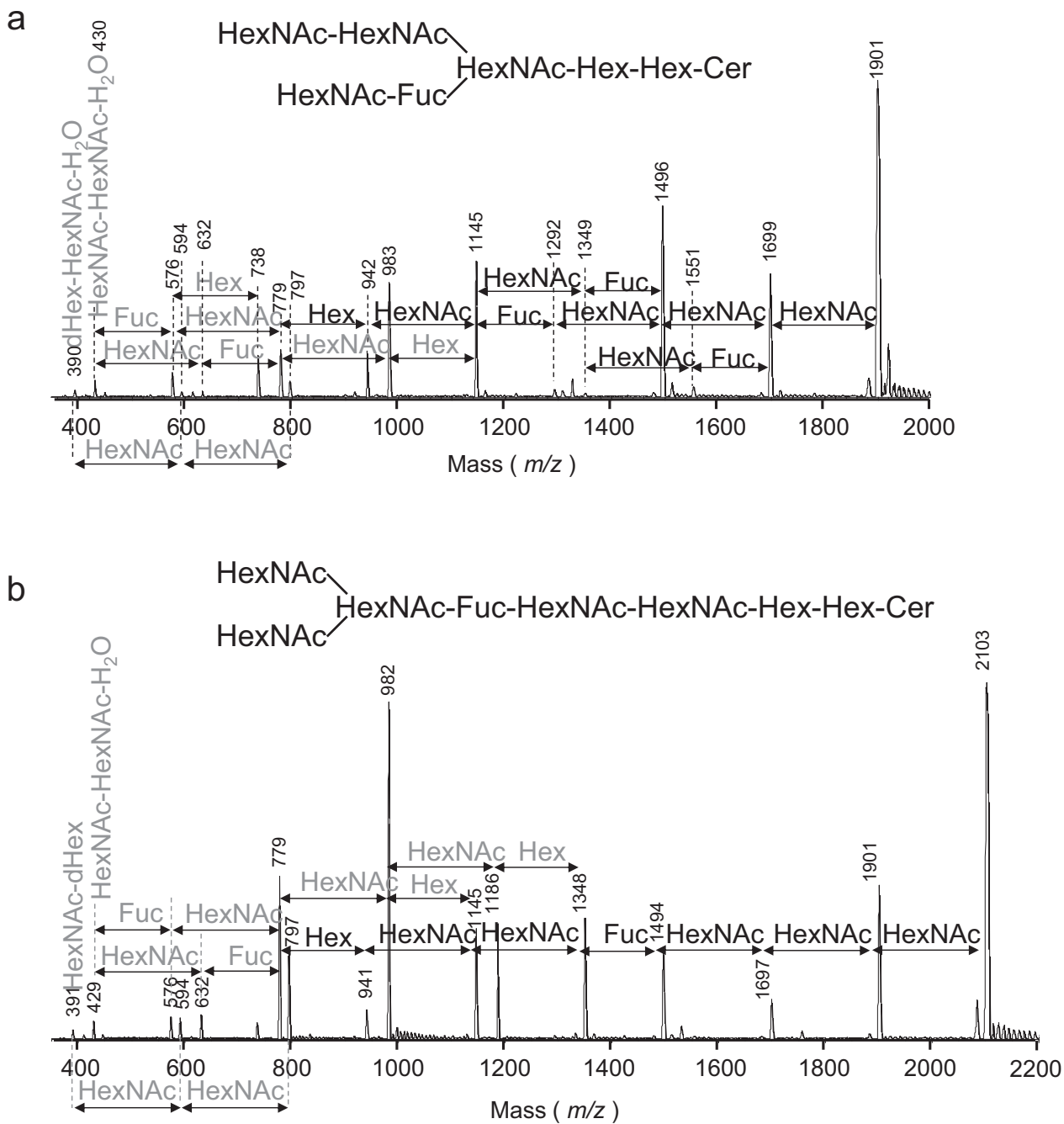

Supplemental fig. 1

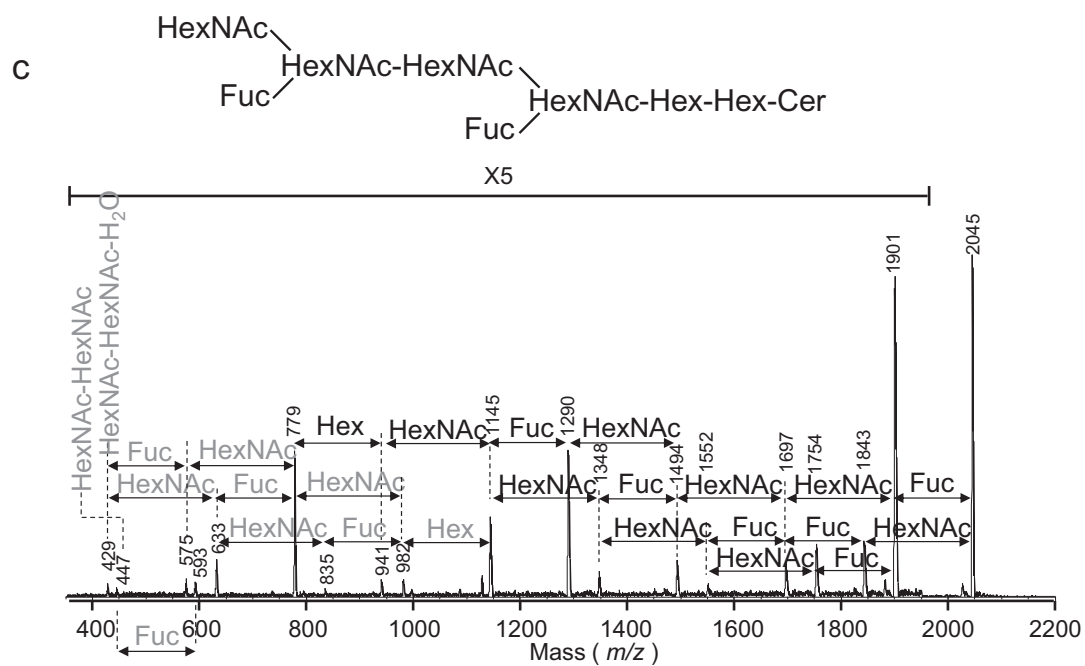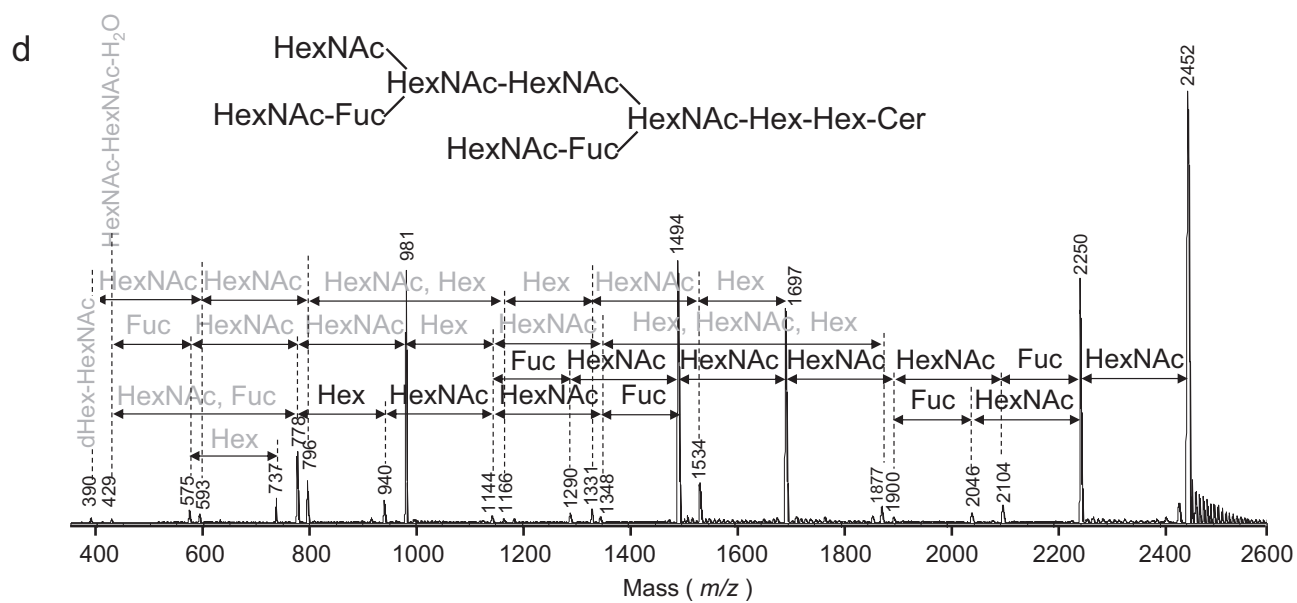

Supplemental fig. 1 (Cont.)

Supplement: Supplementary file 1 — Positive-ion PSD spectra in MALDI-TOF MS of the separated GSLs. (a) CHpS2; (b) COS1; (c) COS2; and (d) CDeS. (PDF 115 kb) [file 10719_2012_9436_MOESM1_ESM.pdf]
