# Supplementary material for: Biochemical studies on sphingolipids of Artemia franciscana: complex neutral glycosphingolipids
Source: Glycoconj J. 2012 Aug 14;30(3):257–68. doi: 10.1007/s10719-012-9436-8 (PMC3606520; doi:10.1007/s10719-012-9436-8)
Supplement: Supplementary file 2 — Summary of MALDI-TOF MS spectra obtained for complex GSLs purified from A. franciscana. Symbols A and B, denoted in the left column, show predominant peaks mentioned in Fig. 3. % distribution was calculated using % value of aliphatic component analysis. tr: trace (PDF 49 kb) [file 10719_2012_9436_MOESM2_ESM.pdf]

Supplemental table (Not to be Published)

Supplemental Table Summary of MALDI-TOF MS spectra obtained for complex GSLs purified from *A. franciscana.* Symbols A and B, denoted in the left column, show predominant peaks mentioned in Fig. 3. % distribution was calculated using % value of aliphatic component analysis. tr: trace

		$CHpS_1$			$CHpS_2$			COS_1		
	FA - LCB	Calc.	Found	% distribution	Calc.	Found	% distribution	Calc.	Found	% distribution
	16:0 - d16:1	1814.95			1814.95			2018.03		
	16:0 - d17:1	1828.97			1828.97			2032.05		
	18:0 - d16:1	1842.98	1843.27	2.5	1842.98	1842.72	3.9	2046.06	2045.74	3.2
	18:0 - d17:1	1857.00	1858.35	1.2	1857.00	1857.74	1.7	2060.08	2059.78	1.6
	20:0 - d16:1	1871.01	1872.38	1.0	1871.01	1871.77	1.4	2074.09	2073.80	1.2
	20:0 - d17:1	1885.03	1885.43	) 0.5	1885.03	1884.81	) 1.4	2088.11	2087.82	) 1.3
	21:0 - d16:1	1885.03	1885.43		1885.03	1884.81		2088.11	2087.82	
	22:1 - d16:1	1897.03	1897.50	2.8	1897.03	1896.85	2.6	2100.11	2099.85	2.5
A	22:0 - d16:1	1899.05	1899.47	56.2	1899.05	1898.82	58.5	2102.13	2101.84	54.0
	22:1 - d17:1	1911.05	1911.42	1.4	1911.05	1910.78	1.2	2114.13	2113.81	1.3
B	22:0 - d17:1	1913.06	1913.42	) 29.0	1913.06	1912.78	) 26.7	2116.14	2115.81	) 29.0
	23:0 - d16:1	1913.06	1913.42		1913.06	1912.78		2116.14	2115.81	
	24:1 - d16:1	1925.06	1925.36	0.7	1925.06	1924.73	tr	2128.14	2129.77	0.9
	23:0 - d17:1	1927.08	1927.36	) 2.4	1927.08	1926.74	) 1.7	2130.16	2131.76	) 2.1
	24:0 - d16:1	1927.08	1927.36		1927.08	1926.74		2130.16	2131.76	
	24:1 - d17:1	1939.08	1939.38	0.3	1939.08	1938.76	tr	2142.16	2143.77	0.5
	24:0 - d17:1	1941.09	1941.39	1.0	1941.09	1940.76	0.6	2144.17	2145.77	0.9

		COS_2		CNS		CDeS		
	FA - LCB	Calc.	Found	Calc.	Found	Calc.	Found	% distribution
	16:0 - d16:1	1961.01		2164.09		2367.17	2367.32	2.6
	16:0 - d17:1	1975.03		2178.10		2381.18	2381.37	1.5
	18:1 - d16:1	1987.03		2190.10		2393.18	2393.38	2.1
	18:0 - d16:1	1989.04	1988.89	2192.12	2192.24	2395.20	2395.39	3.6
	18:1 - d17:1	2001.04		2204.12		2407.20	2407.36	1.3
	18:0 - d17:1	2003.06	2002.92	2206.14	2206.27	2409.22	2409.40	2.2
	20:0 - d16:1	2017.07	2016.92	2220.15	2220.27	2423.23	2423.41	1.3
	20:0 - d17:1	2031.09	2030.95	2234.17	2234.33	2437.25	2437.44	) 1.4
	21:0 - d16:1	2031.09	2030.95	2234.17	2234.33	2437.25	2437.44	
	22:1 - d16:1	2043.09	2042.95	2246.17	2246.43	2449.25	2449.46	0.7
A	22:0 - d16:1	2045.10	2044.96	2248.18	2248.38	2451.26	2451.45	49.5
	22:1 - d17:1	2057.10	2056.95	2260.18	2260.29	2463.26	2463.42	0.4
B	22:0 - d17:1	2059.12	2058.95	2262.20	2262.31	2465.28	2465.42	) 30.6
	23:0 - d16:1	2059.12	2058.95	2262.20	2262.31	2465.28	2465.42	
	24:1 - d16:1	2071.12	2072.95	2274.20	2274.17	2477.28		
	23:0 - d17:1	2073.14	2074.93	2276.21	2276.24	2479.29	2479.39	) 1.7
	24:0 - d16:1	2073.14	2074.93	2276.21	2276.24	2479.29	2479.39	
	24:1 - d17:1	2085.14		2288.21		2491.29		
	24:0 - d17:1	2087.15		2290.23	2290.29	2493.31	2493.41	0.8
